# Secondary Amyloidosis and Common Variable Immunodeficiency: A Rare Association

**DOI:** 10.7759/cureus.31976

**Published:** 2022-11-28

**Authors:** Joana Lopes, Maurício Peixoto, Eulália Antunes, Isabel Silva, Sofia Caridade

**Affiliations:** 1 Internal Medicine, Hospital de Braga, Braga, PRT; 2 Oncology, Hospital de Braga, Braga, PRT

**Keywords:** common variable immunodeficiency, nephrotic syndrome, rapidly progressive renal failure, hypogammaglobulinemia, secondary amyloidosis

## Abstract

Common variable immunodeficiency (CVID) is a disease characterized by severe antibody deficiency due to impaired B cell differentiation. It represents the most common form of primary immunodeficiency in children and adults, and its clinical manifestations include recurrent infections and chronic lung disease, gastrointestinal infections, and autoimmunity. Here, we present the case of a 47-year-old female patient with a history of CVID and recurrent *Campylobacter jejuni* bacteremia. She was undergoing biweekly administration of intravenous immunoglobulin for over 15 years. During hospitalization rapidly progressive oliguric renal failure was observed in association with anasarca and nephrotic syndrome. Bilateral nephromegaly was noted on an abdominal pelvic computed tomography scan. Renal biopsy was consistent with amyloidosis, and serum amyloid A protein was elevated. The diagnosis of AA amyloidosis secondary to CVID was made. The patient was started on hemodialysis and weekly intravenous immunoglobulin administration with favorable clinical outcomes.

## Introduction

Common variable immunodeficiency (CVID) is a disease characterized by severe antibody deficiency due to impaired B cell differentiation which leads to hypogammaglobulinemia. It represents the most common form of severe antibody deficiency with the age at diagnosis ranging from the third to fifth decades of life, with late diagnosis being common [1,2]. The clinical manifestations include recurrent infections and chronic lung disease, gastrointestinal infections, autoimmunity, and even malignancy. The treatment consists of lifelong immunoglobulin (Ig) replacement therapy to diminish the risk of future infections [3]. Secondary amyloidosis is a rare complication of CVID, with only a few published case reports [2].

Here, we present the case of a young female patient with a history of CVID and biweekly Ig administration, spanning over 15 years, who developed renal amyloidosis. We also review previously published relevant cases and the clinical presentation. Our objective is to highlight the importance of considering secondary amyloidosis as a potential, albeit rare, complication of CVID, and the need for reassessing the frequency of Ig repositioning to control chronic immunosuppression in these patients.

## Case presentation

A 47-year-old female patient undergoing treatment for an ischemic stroke and spontaneous bacterial peritonitis was referred from another department due to rapidly progressive renal failure. Her medical history included CVID with biweekly intravenous Ig administration for over 15 years. She also had chronic liver disease of unknown etiology and recurrent *Campylobacter jejuni *bacteremia. About three months prior to her current admission her renal function was normal. On admission, she was under low-flux oxygen, hemodynamically stable, and apyretic. Physical examination revealed anasarca with jugular vein distention and a systolic heart murmur. She was oliguric and had acute kidney injury, erythrocyturia, and nephrotic-range proteinuria. Her other laboratory studies are presented in Table 1.

**Table 1:** Laboratory results.

Parameter	Value	Reference values
Hemoglobin (g/dL)	10	11.9–15.6
Hematocrit (%)	31.6	36.6–45
Mean corpuscular volume (fL)	85.9	82.9–98
Mean corpuscular hemoglobin (pg)	27.2	27–32.3
Leukocytes (/μL)	12,300	4,000–11,000
Platelets (/μL)	276,000	150,000–450,000
Urea (mg/dL)	251	19–49
Creatinine (mg/dL)	3.5	0.6–1.2
Sodium (mmol/L)	140	135–145
Potassium (mmol/L)	4.6	3.5–5.1
Total protein (g/dL)	4.3	5.7–8.2
Albumin (g/dL)	2.2	3.4–5.0
C-reactive protein (mg/L)	33.10	<5.0
IgA (mg/dL)	<15	40–350
IgG (mg/dL)	561	650–1,600
IgM (mg/dL)	<8	50–300
C3 (mg/dL)	130	90–180
C4 (mg/dL)	35	10–40
Direct and indirect Coombs test	Negative	
Transferrin saturation (%)	11.9%	20–45
24-hour urinary protein (mg)	3,500	<150

**Figure 1:**
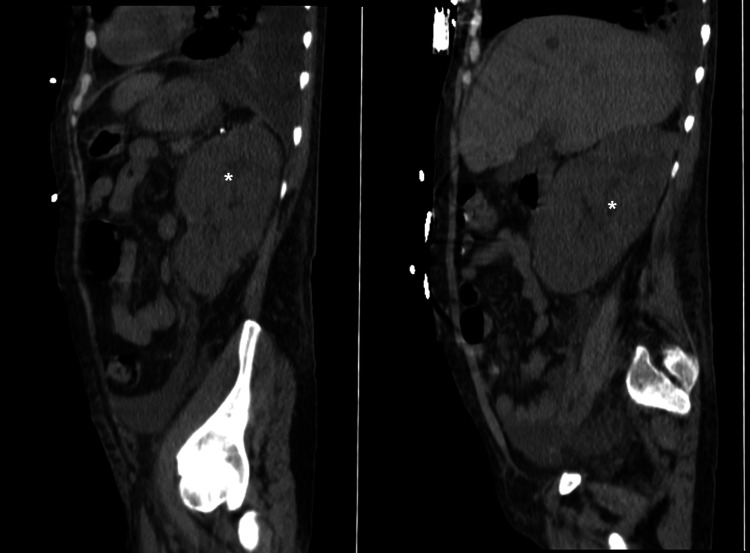
Bilateral nephromegaly (*) with a 170 mm long bipolar axis.

Transferrin saturation was compatible with iron deficiency. Serum protein electrophoresis, immunoelectrophoresis, and serum and urinary light chain ratio were normal. Infectious serologic tests were normal for HIV I/II, hepatitis B and C, herpes simplex virus 1 and 2, cytomegalovirus, and Epstein-Barr virus. The autoimmunity panel, including anti-ds DNA antibodies, anti-neutrophil antibodies, anti-nuclear antibodies, anti-glomerular basement membrane antibodies, cryoglobulins, and circulating immune complexes, was also normal. Abdominal and pelvic computerized tomography (CT) scan revealed bilateral nephromegaly, with a 170 mm long bipolar axis, enlargement of the left hepatic lobe, and presence of large-volume ascites (Figure 1).

Transthoracic echocardiogram showed slight left atrial dilation, normal global systolic function, and mild tricuspid insufficiency. No vegetations were detected. As the patient had a combination of rapidly progressive renal failure with nephrotic syndrome, in association with a background of recurrent systemic infections due to an immunodeficient state, the possibility of secondary renal amyloidosis was considered. A renal biopsy was performed and was compatible with tubulointerstitial nephritis associated with amyloidosis. Immunofluorescence tests were negative for IgM, IgG, IgA, C1q, C3c, or lambda and kappa light chains. Histochemical tests with Congo red staining revealed amyloid substance deposition in the glomeruli, blood vessels, and tubules. Dosing of serum A amyloid was 5.7 mg/L (high). A diagnosis of secondary renal amyloidosis was formulated.

During hospitalization and due to refractory hypervolemia, the patient was put on a jugular venous central line and started a regular hemodialysis program. There was a need for weekly Ig administration to maintain normal serum levels. Her outcome was favorable but hemodialysis was continued in a programmed regimen after discharge.

## Discussion

Amyloidosis is a group of disorders associated with the deposition of abnormal protein fibrils with resulting organ damage. There are several types of systemic amyloidotic syndromes, with the main ones being light-chain amyloidosis (AL), secondary amyloidosis (AA), hemodialysis-related amyloidosis (Aβ2M), and transthyretin amyloidosis (ATTR), both familial (mutant) and age-related (wild-type) [4].

The development of secondary amyloidosis stems from the continuous accumulation of serum amyloid A (SAA), a high-density apolipoprotein produced in the hepatocytes in response to pro-inflammatory stimuli by cytokines such as interleukin (IL)-6, IL-1, and tumor necrosis factor-alpha 6 [5]. Its exact prevalence in patients with inflammatory diseases is difficult to assess as its diagnosis depends on numerous factors, such as the location of the biopsy and clinical manifestations versus asymptomatic amyloid deposition. There have been estimates in some studies with patients suffering from rheumatoid arthritis and familial Mediterranean fever, but with ongoing advances in anti-inflammatory and biologic therapies along with an early diagnosis of these pathologies, these estimates have been modified [6]. A 2011 nationwide series in Finland showed an important decline in the incidence of renal replacement therapies due to secondary amyloidosis in patients with chronic inflammatory diseases who were being treated with disease-modifying anti-rheumatic drugs and biologic therapies [7].

Secondary amyloidosis can occur in response to any chronic inflammatory state, with 40% of cases related to rheumatoid arthritis [4]. Other known causes include periodic fever syndromes, such as familial Mediterranean fever, inflammatory bowel disease, lymphoproliferative disorders, and tuberculosis, but very few cases have been described in association with CVID or other hypogammaglobulinemia in the last 25 years [1-4,8-16].

The most commonly affected organ is the kidney, with asymptomatic proteinuria, progression to nephrotic syndrome, and kidney failure representing the most common and earliest forms of presentation [5,9,13]. Of the 12 reported cases of secondary amyloidosis, eight had renal involvement, five had gastrointestinal involvement, and one had pulmonary involvement, as shown in Table 2 [1-3,9,11-14,16].

**Table 2:** Main secondary amyloidosis cases of patients suffering from common variable immunodeficiency disorder, hypogammaglobulinemia, and agammaglobulinemia spanning the last 25 years. CVID: common variable immunodeficiency

Authors	Gender and age	Underlying chronic disorder	Organs with amyloid deposition
Meira et al. (2015) [8]	Female 66 years old	Rheumatoid arthritis and CVID	Gastrointestinal
Esenboga et al. (2015) [1]	Male 27 years old	CVID	Renal
Balwani et al. (2015) [9]	Male 40 years old	Pulmonary tuberculosis and CVID	Renal
Arslan et al. (2015) [2]	Male 24 years old	CVID	Pulmonary and renal
Borte et al. (2014) [10]	Female 20 years old	CVID	Gastrointestinal
Kadiroğlu et al. (2012) [11]	Female 24 years old	CVID	Renal
Firinu et al. (2011) [3]	Female 66 years old	CVID	Renal
Aydin et al. (2010) [12]	Female 29 years old	CVID	Renal
Aghamohammadi et al. (2010) [13]	Male 50 years old	CVID	Renal
Çelik et al. (2005) [14]	Male 28 years old	CVID	Gastrointestinal
Kotilainen et al. (1996) [16]	Female 49 years old	CVID	Renal and gastrointestinal
Tezcan et al. (1998) [15]	Male 27 years old	X-linked agammaglobulinemia	Gastrointestinal

Ongoing asymptomatic proteinuria was already documented in our patient before the onset of nephrotic syndrome and acute kidney injury with the need for dialysis. Regarding the usual age of onset, 11 out of 12 of these patients fit in the third to fifth decades of life, as did our patient.

The most probable cause surrounding CVID as a trigger to the development of renal amyloidosis was believed to be her history of recurrent *Campylobacter jejuni* bacteremia prior to this admission. According to a recent review article by Okuda, the most important parameter in the treatment of secondary amyloidosis is the inhibition of the continuous activity of the underlying inflammatory stimuli, with SAA concentrations being directly related to prognosis. Our patient’s SAA dosing revealed a value of 5.7 mg/L, which amounts to a 10-year survival rate of about 90%, according to recent literature [5]. Albeit undergoing biweekly immunoglobulin administration, the patient maintained a history of persistent and frequent infections. To prevent future events after clinical compensation during hospitalization, weekly Ig administration was established with favorable outcomes.

## Conclusions

Although very rare, secondary amyloidosis is a diagnosis to consider when faced with rapidly progressive renal failure in a patient with a chronic inflammatory status. CVID is an important source of prolonged exposure to inflammatory stimuli, such as infections and autoimmune disorders, with a late diagnosis at times and consequent delay in treatment. This case also highlights the importance of reassessing the frequency of Ig repositioning in patients with hypogammaglobulinemia as a way of controlling SAA concentrations and the consequences of immunosuppression.
